# Symmetric square shaped metamaterial structure with quintuple resonance frequencies for S, C, X and Ku band applications

**DOI:** 10.1038/s41598-021-83715-x

**Published:** 2021-02-19

**Authors:** Tayaallen Ramachandran, Mohammad Rashed Iqbal Faruque, Mohammad Tariqul Islam

**Affiliations:** 1 Space Science Center (ANGKASA), Universiti Kebangsaan Malaysia, 43600 UKM Bangi, Selangor Malaysia; 2 Department of Electrical, Electronic and Systems Engineering, Universiti Kebangsaan Malaysia, 43600 UKM Bangi, Selangor Malaysia

**Keywords:** Electrical and electronic engineering, Electronic properties and materials

## Abstract

This study explores the effect of symmetrical square shaped metamaterial design for microwave frequency applications. The latest technology demands of advanced performance and research studies of metamaterial integration in the related bands are increasing tremendously. Therefore, this motivates us to explore the metamaterial design structure that has a high possibility to be applied in more than two resonance bands using a compact design structure. This study emphasis on a compact 14 × 14 mm^2^ and 1.524 mm thick substrate material known as Rogers RT6002. Seven distinct square shaped metamaterial (SQM) rings were constructed on the substrate material to achieve the goal of this research study. Besides that, the investigations of the metamaterial electromagnetic properties and effective medium parameters were carried out by utilising the Computer Simulation Technology Microwave Studio (CST) software. According to the numerical simulation results, the proposed SQM unit cell manifested quintuple resonance frequencies precisely at 3.384 (S band), 5.436, 7.002 (C band), 11.664 (X band), and 17.838 GHz (Ku band). Meanwhile, for the validation process, the comparison between the simulation and measurement results was analysed and data showed that the first and third resonance frequencies were increased by 0.336 and 0.139 GHz, respectively while other frequencies were reduced by 0.186, 0.081, and 0.709 GHz in sequential order. The numerical simulation of the metamaterial design was conducted in a High Frequency Structure Simulator (HFSS) to further validate the results. Furthermore, the proposed SQM manifested left handed characteristics at the second to fifth resonance bands. In a nutshell, the SQM successfully achieves the objectives of this research work and can be applied to multi band applications.

## Introduction

A material that possesses unusual and unique performance, known as a metamaterial, is becoming a hot topic among scientists. This artificially-structured material is not found in nature and has superior electromagnetic properties that are hard to obtain in conventional materials. Besides that, the metamaterial possesses unexpected ways of interaction between light and sound waves. The light propagation can be controlled by this man-made material in waveguides and free space. Metamaterials have been utilised in a wide range of application fields, for instance, sensor, Specific Absorption Rate (SAR) reduction, satellite application, terahertz frequency application, superconductor, optical cloaking, microwave application, etc.^1–4^. Nevertheless, these series of recent studies have indicated that the application fields are different from each other, and the distinctive properties of the metamaterial allowed the researchers to obtain remarkable results. Generally, the metamaterial is not particularly new and has been in existence for many years. The unique characteristic, i.e., left-handed metamaterial has been adopted in the recent research studies to optimise the performance. The left-handed metamaterial possesses negative behaviour in permittivity, permeability, and refractive index. However, it seems to be a common problem in gaining the left-handed characteristic for all the resonance frequencies with a distinct metamaterial design.

Previous research studies suggest that the metamaterial can influence the outcome in the satellite application field. In 2020, Tayaallen et al.^5^ proposed a dual-band novel combination of circular and square-shaped left-handed metamaterial for satellite application. The authors successfully developed the metamaterial design on the Epoxy Resin Fibre (FR-4) substrate material, which manifested double resonance frequencies in the range of 0–18 GHz. A compact planar metamaterial-based antenna for satellite application was suggested by Subhash et al.^6^ in 2018. They designed a compact 17 × 17 mm^2^ metamaterial antenna that consisted of a regular decagonal split ring resonator as a radiating part and a feed line. A good impedance matching and stable radiation pattern were successfully achieved in their study, implying that the suggested design is suitable for satellite application. Meanwhile, Parul et al.^7^ introduced a metamaterial-inspired patch antenna with a two-segment split ring resonator Labyrinth embedded inside the antenna substrate. The application of the study focused on a single band resonance frequency to improve the voltage standing wave ratio. Meanwhile, Sikder et al.^8^ presented two component analyses of negative index metamaterial for C- and X-bands applications. Sikder et al. designed the metamaterial structure on epoxy resin composite with woven glass fibre and the simulation was successfully carried out using the CST software.

Many existing studies in the broader literature have examined microwave applications by utilising the unconventional material. Metamaterial in microwave application has become a hot topic among researchers in the past few years. A double negative characteristic with modified split H-shaped metamaterial was designed and analysed by Sikder et al.^9^ in 2020. The z-axis wave propagation and Rogers RO3010 substrate material were utilised in this investigation and focused on C-band applications. Meanwhile, the same author^10^ investigated the performance changes of the metamaterial by using two substrate materials, namely FR-4 and Rogers RT6010. The proposed unit cell metamaterial manifested negative permittivity or double negative characteristics for each substrate material, accordingly. Faruque et al.^11^ investigated a flexible metamaterial design for microwave application. The authors successfully developed and designed metamaterial structure on a flexible substrate material known as nickel aluminate which exhibited double resonance bands. Meanwhile, Ikbal et al.^12^ proposed a wide-band double negative metamaterial for C- and S-band applications. This study explored the frequency range from 0.5 to 7 GHz and simulated by utilising finite-different time-domain method based on CST software.

This paragraph presents a review of recent literature on metamaterial absorption. A metamaterial absorber is a type of metamaterial designed to properly absorb electromagnetic radiation such as light. Zilong et al.^13^ proposed a multi-layered absorber based on magnetic material. This study focused mainly on the lower frequency range from 2 to 4 GHz for broad-band absorption. The multi-layered absorber comprised of metamaterial structures and magnetic coatings that were stacked layer by layer. In 2015, Mayank et al.^14^ designed a compact metamaterial absorber for X-band application. The proposed metamaterial consisted of a square patch enclosed within a square-closed ring resonator and connected by a tilted strip. Whereas Prarthan et al.^15^ presented a novel wideband metamaterial absorber for S-, C-, and X-band applications. This investigation successfully designed, analysed, and simulated the wideband metamaterial absorber in Ansoft HFSS software. Two C-shaped metamaterial structures were placed diagonally opposite to each other on the FR-4 substrate material. Meanwhile, Wang et al. carried out investigations on the terahertz metamaterial absorber in 2017 and 2020^16,17^. These studies mainly focused on the absorber for dual- and quad-band applications. Moreover, the authors proposed two types of metamaterial design structures for specific applications, which were a common sandwich structure and two identical square metallic patches, respectively. The focus of the research work^16^ revealed a quad-band absorber application only while reference^17^ investigated the terahertz absorber for sensing application.

The previous studies that stated in the literature review have significant contribution in many application fields; however, emerging latest technologies demand an advanced method or design to further optimise the performance. Therefore, we argue that the previous works of literature suffer from certain weaknesses. The number of questions regarding the size of substrate material, unique characteristics of the metamaterial, and number of resonance frequencies and square rings need to be taken into consideration. As mentioned earlier, there has been a great deal of difficulty when constructing the metamaterial design structures that need to obey all constraints listed above. Most of the previous studies have solitary special characteristic and effectively generate multi resonance frequencies but the works are limited by the bigger size of array metamaterial structures. Meanwhile, the initial study of this present work demonstrates that the left-handed characteristic is not possible for all kinds of design structure. Besides that, the number of rings in the metamaterial design acts as a key factor in generating multi-band resonance frequencies and left-handed characteristics. Hence, the focus of this paper is to demonstrate a metamaterial design with quintuple resonance frequencies and left-handed characteristics while maintaining the array size below average level.

## Materials and methods

### SQM unit cell construction

**Figure 1:**
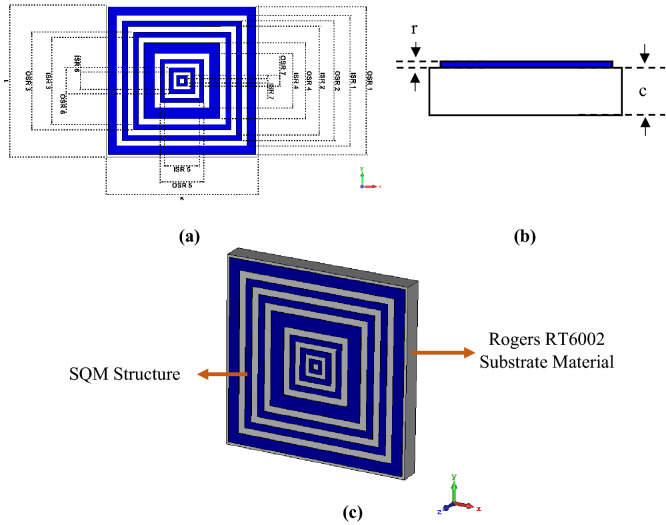
SQM design from CST software: (**a**) top view, (**b**) front view, (**c**) isometric projection view.

Figure 1a–c illustrate the completed SQM design at several viewpoints that were exported from CST software. The full dimension of the metamaterial design structure is demonstrated in Fig. 1a. The unit cell of the proposed SQM was originally composed of Rogers RT6002 dielectric substrate material of length, t = 14 mm and width, s = 14 mm. The substrate thickness, c = 1.524 mm as shown in Fig. 1b was adopted in this investigation. During the selection of the precise substrate material, the tangent loss (δ = 0.0012) and dielectric constant (ε = 2.94) of the RT6002 material were considered. The substrate material has excellent dimensional stability and low outgassing which are ideal for space applications. Besides that, this type of material has a low loss for excellent high-frequency performance. This work is based on exactly seven square rings with specific gaps and thickness that were constructed on the selected substrate material. For the development of the SQM design, annealed copper material with conductivity, σ = 5.80 × 10^7^ S/m was adapted, and the thickness was finalized at 0.035 mm from the surface of RT6002. The first square ring near to the positive x-axis was constructed with an outer (OSR) and inner square (ISR) design of 13.60 mm and 12.00 mm length, respectively. The ISR then subtracted from the OSR structure to produce the first square ring. Subsequently, with a gap of 1.0 mm, the second ring was built with a width of 1.0 mm. Furthermore, the first 5 rings have similar gaps between them and almost equivalent ring width except rings 4 and 5 with 2.0 mm and 0.8 mm, respectively. The next two rings have gaps reduced gradually about 0.2 mm from 1.0 mm for each ring. Meanwhile, the widths of these last two rings were fixed at 0.8 mm and 0.60 mm, respectively. These rings were designed as such to gain multi-resonance frequencies that are applicable for many applications. The isometric projection view of the proposed SQM is demonstrated in Fig. 1c. Table 1 describes the dimension lists of the SQM unit cell design.

**Table 1:** Description of the proposed SQM design.

Descriptions	Dimension (mm)
OSR 1	13.60
ISR 1	12.00
OSR 2	11.00
ISR2	10.00
OSR 3	9.00
ISR3	8.00
OSR 4	7.00
ISR4	5.00
OSR 5	4.00
ISR5	3.20
OSR 6	2.40
ISR6	1.60
OSR 7	1.00
ISR7	0.40
RT6002 length, t	14.00
RT6002 width, s	14.00
Metamaterial thickness, r	0.035
RT6002 thickness, c	1.524

*OSR *Outer square dimension of square ring, *ISR *inner square dimension of square ring.

### Numerical simulation method

**Figure 2:**
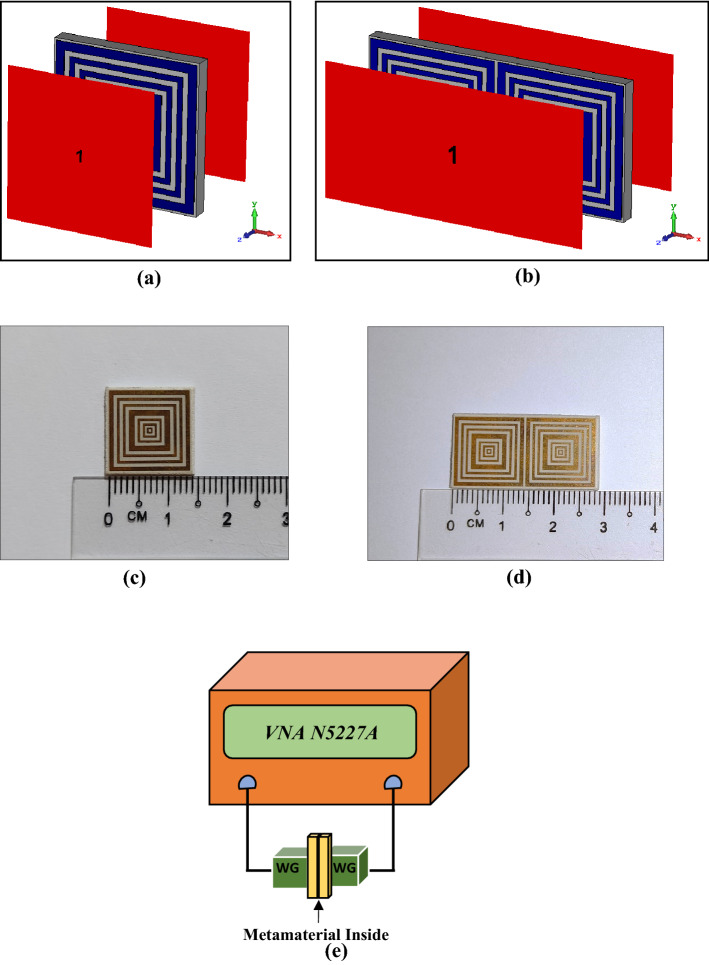
Numerical simulation geometry and fabrication measurement setup: (**a**) SQM unit cell simulation, (**b**) 1 × 2 SQM array simulation, (**c**) fabricated SQM unit cell, (**d**) fabricated 1 × 2 SQM array, (**e**) metamaterial measurement setup of VNA N5227A.

Numerical simulations were performed with the help of a familiar software known as Computer Simulation Technology (CST) Microwave Studio. Typically, CST is used in electromagnetic problem solving that has precise and quick responses. Besides that, CST software provides significant product-to-market advantages such as shorter development cycles or virtual prototyping before physical trials. Meanwhile, the High-Frequency Structure Simulator (HFSS) software was adopted to validate the numerical simulation results. This method attempts to solve the problem following a four stage process, namely unit cell analysis, array cell analysis (further details about array cell selection in “Metamaterial array structure analysis”), effective medium parameter analysis, and validation procedure. The first two stages were numerically performed by utilising frequency-domain solver and tetrahedral mesh in CST software. The frequency-domain solver is known as a powerful all-purpose 3D full-wave solver that offers brilliant simulation performance. This solver can calculate all the ports at the same time and, therefore, it is suitable to simulate multi-port systems, for instance, array structures. Both SQM unit and array cells were fixed at the middle of two waveguide ports as shown in Fig. 2a,b. Meanwhile, the ports were positioned at positive and negative z-axis by utilising a transverse electromagnetic wave. This wave mode possesses neither electric nor magnetic field in the direction of propagation. Subsequently, a perfect electric conductor boundary condition was set for the x-axis, while the y-axis was set as a perfect magnetic conductor. As this study aims to encompass several resonance bands, the frequency range was set from 0 to 18 GHz. The primary objective of this simulation was to calculate the scattering parameters of the proposed unit and array cells metamaterial. Then, the collected reflection and transmission coefficient results of the SQM array cell were exploited to calculate the effective medium parameters, i.e., permittivity (ε), permeability (μ), refractive index (n), and impedance (z) values. The well-known Robust method was applied for this computation to identify the unique electromagnetic properties of the SQM using MATLAB software^18–20^. This multi-paradigm numerical computing has an easy-to-use environment and is proficient in resolving mathematical notation problems. The retrieval equations of the impedance ($z$), refractive index ($n$), permittivity (ε), and permeability (μ) values are defined in Eqs. (1) to (4). For the final stage, the scattering parameters of the SQM unit cell design was validated using the HFSS software by adopting similar methods and techniques as in CST.

1 $$ z = \pm \surd \frac{{\left( {1 + S_{11} } \right)^{2} - S_{21}^{2} }}{{\left( {1 - S_{11} } \right)^{2} - S_{21}^{2} }}, $$

$$ n = \frac{1}{{k_{0} d}}\left\{ {\left[ {In\left( {e^{{ink_{0} d}} } \right)} \right]^{\prime \prime } + 2m\pi - i\left[ {In\left( {e^{{ink_{0} d}} } \right)} \right]^{\prime } } \right\}, $$

2 $$ e^{{ink_{0} d}} = \frac{{S_{21} }}{{1 - S_{11} \frac{z - 1}{{z + 1}}}}, $$

3 $$ \varepsilon = \frac{n}{z}, $$

4 $$ \mu = nz, $$

### Measurement process

The scattering parameters of the unit and array cells were obtained through both numerical and measurement processes. The unit and array cells of SQM were fabricated on a printed circuit board as indicated in Fig. 2c,d, to calculate the scattering parameters experimentally. In general, the boards are built with copper material placed on the surface of Rogers RT6002 material. Based on the proposed SQM structure, the excessive copper on the substrate was carved away. The measurement process for this fabricated metamaterial was carried out using a vector network analyser (VNA) model number of Agilent N5227A (experimental setup illustrated in Fig. 2e). The unit and array cells of the SQM were placed in the middle of both waveguide ports that were connected to the VNA measuring instrument. Four types of adapters were utilised in this process, namely A-INFOMW WG to Coaxial adapter P/N: 187WCAS for S-band, A-INFOMW WG to adapter P/N: 137WCAS for C-band, A-INFOMW WG to adapter P/N: 112WCAS for X-band, and A-INFOMW WG to adapter P/N:51WCAS-CU for Ku-band. Under certain circumstances, an error might occur in the hardware during the measurement process; thus, to ensure accurate outcomes, an Agilent N4694-60001 instrument kit was used to calibrate the VNA before the measurement began.

## Results and discussion

**Figure 3:**
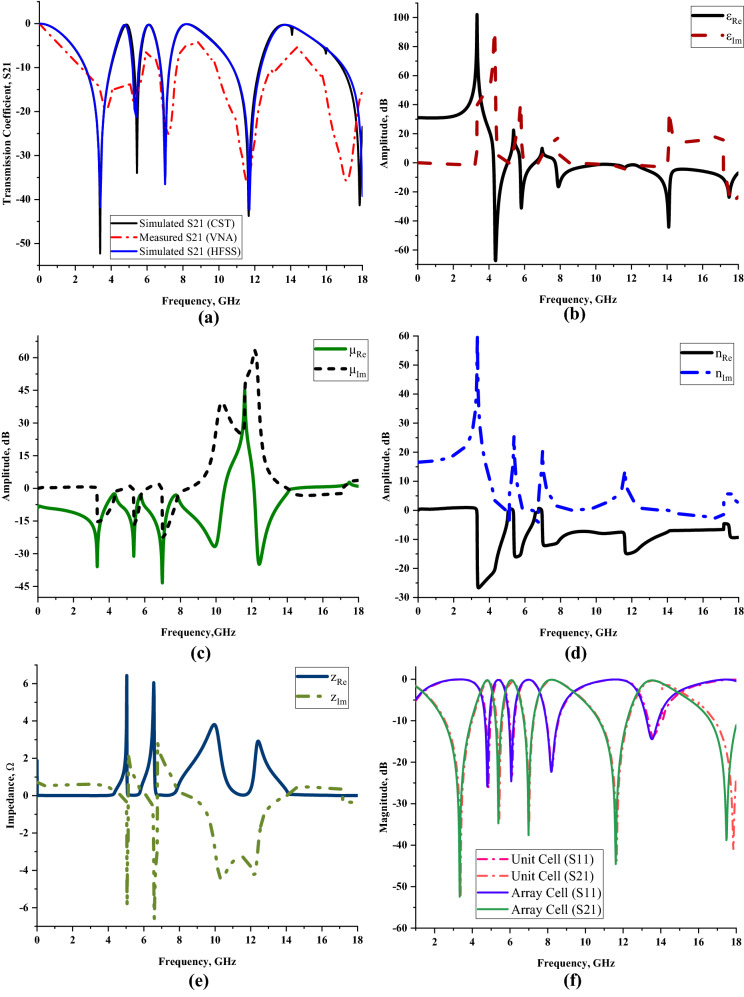
Scattering and effective medium parameters of the proposed SQM design: (**a**) Transmission coefficient (unit cell) of CST, VNA and HFSS, (**b**) permittivity, (**c**) permeability, (**d**) refractive index, (**e**) impedance value; (**f**) comparison of numerically simulated unit and array cells (S11 and S21).

Figure 3a–f illustrate the results collected from the numerical simulation in terms of the transmission and reflection coefficients, permittivity, permeability, refractive index, and impedance values. Our findings in Fig. 3a demonstrates that the quintuple resonance frequencies of unit cell simulation on z-axis wave propagation at S-, C-, X- and Ku-bands were 3.384, 5.436, 7.002, 11.664, and 17.838 GHz with tolerable magnitude values of − 52.297, − 34.745, − 33.977, − 43.756, and − 41.336 dB, respectively. The transmission coefficient measurement results of the SQM were also compared in Fig. 3a which shows slightly non-identical resonance frequencies of 3.720, 5.250, 7.141, 11.583, and 17.129 GHz, respectively. The measurement data shows acceptable magnitude values such as − 19.600, − 17.700, − 25.100, − 36.400, and − 35.800 dB. Overall, there was a mean difference of 4.001% between the numerical and measured transmission coefficient results of the SQM unit cell for all resonance frequencies.

Although the results indicate a negligible difference, the discrepancies between these two methods may be caused by several parameters. First, the calibration error might influence the measurement results. The Agilent N5227A instrument underwent a calibration process before the experiment was carried out. An Agilent N4694-60001Ecal calibration kit was utilised in this investigation, which helped to obtain accurate measurement results due to the ambient temperature effects. These effects are known as drift errors and occurred due to the performance change in the instrument after the calibration process. The variation in temperature affects the instrument to produce invalid outcomes. The stable ambient temperature during the measurement process will help minimize the effects. Therefore, a stabilised, well warmed-up, and ventilated instrument is necessary for this process. Moreover, minor errors that happen during the fabrication process probably exhibit distinguishable measured results. As the proposed SQM is compact in size, accidental flaws are possible when designing copper metamaterial on the Rogers RT6002 material. Furthermore, the inevitable variation in the simulated and measured results is caused by the mutual resonance effect that exists between the two waveguide ports. Meanwhile, the transmission coefficient results that were computed using HFSS software are demonstrated in Fig. 3a. A total difference average value of 0.476% in frequency was produced through the HFSS method. As the discrepancy of the results between CST and HFSS method is less than 5%, then it can be acceptable for validation purposes. Besides that, the numerically simulated transmission and reflection coefficient results, illustrated in Fig. 3f, exhibited non-identical findings. The reason behind this phenomenon is because not all the power was reflected or transmitted during the simulation process. Moreover, it provides distinct outcomes based on the metamaterial design structure and the type of substrate material.

The effective medium parameter is a platform to identify the effect of external time-variant electromagnetic fields exposure on the unique material structure. Generally, the connection between the electric and magnetic fields refers to the physical characteristics of the electromagnetic field. This phenomenon occurs in space due to the electric charges which possess time-varying behaviour. Only static charges are produced in space through the static fields. However, the source of time-varying electric charges probably has a high enhancement of the magnetic field and subsequently produces time-variant electric fields. Commonly, most of the materials are divided into two main categories, namely lossy and dispersive materials. These materials usually manifest superior resonance frequencies, unique permittivity, and permeability values.

Our findings on the permittivity values displayed in Fig. 3b clearly reveal five negative permittivity characteristics at C-, X-, and Ku-bands. At the C-band, the frequency ranges of 4.266 to 5.040 GHz, 5.742 to 6.570 GHz, and 7.722 to 7.992 GHz have negative permittivity behaviour with acceptable amplitude values below zero of − 0.494 to − 0.245 dB, − 0.657 to − 0.105 dB, and − 0.231 to − 13.424 dB, respectively. Meanwhile, another two negative permittivity values occurred each at the X- and Ku-bands. For the frequency range from 8.010 to 11.988 GHz, the proposed metamaterial has a negative behaviour with amplitude values from − 12.778 to − 1.441 dB, respectively. Lastly, the frequency range from 12.006 to end possesses negative permittivity values with a peak at 14.094 GHz and an amplitude value of − 44.317 dB. Overall, the highest recorded negative behaviour was at 4.374 GHz with an amplitude value of − 67.413 dB. Additionally, the SQM has four negative permeability ranges at all resonance bands as shown in Fig. 3c. Essentially, frequency range differences of 1.980, 3.978, and 2.466 which started from 2.016, 4.014, and 8.010 GHz have amplitude values that continuously fluctuated from − 11.171 to − 0.320 dB for the first three resonance bands. However, at the Ku-band, negative permeability values were observed from 12.132 up to 14.472 GHz with the amplitude values moderately shifting between − 0.341 and − 0.004 dB.

Another promising highlight from these results is that the metamaterial has a left-handed characteristic for all the resonance bands except the S-band. The fundamental theory of the left-handed characteristic refers to not only the refractive index having negative behaviour, but the permittivity and permeability values also need to be below zero. For example, the frequency ranges of the first two negative permittivity values stated above possessed left-handed characteristic with permeability and refractive index (as illustrated in Fig. 3d) values from − 2.503 to − 11.422 dB, − 20.974 to − 1.717 dB and − 3.138 to − 14.926 dB, − 15.324 to − 1.441 dB, respectively. The other two left-handed frequency ranges fall in three resonance bands. The continuous fluctuation of amplitude values from 7.722 to 10.476 GHz has respective effective medium parameters from − 0.231 to − 1.019 dB, − 3.077 to − 0.320 dB, and − 11.503 to − 7.972 dB. This frequency range falls in both C- and X-bands accordingly. The last left-handed characteristic occurred at the frequency range from 12.132 to 14.472 GHz (Ku-band) which has the second best effective medium parameter performance. Commonly, left-handed metamaterial that possesses unique characteristics is possible to be applied in a broad fields of applications, for instance, sensor, satellite application, radome, SAR reduction, metamaterial antenna, coupler, etc. This is because the left-handed property generally optimises the performance of the material such as exhibiting reversed Doppler shifts, negative refraction index, and propagation in the backward wave. In addition, the electromagnetic metamaterial that comprises a distinct structure that offers a range of response ultimately provides other physical properties, namely elasticity, heat conduction, or strength.

Figure 3e indicates the impedance values that were computed in MATLAB. Four peak impedance values were recorded in this study at three resonance bands. At the C-band, the impedance value reached a maximum point of 6.432 and 6.058 Ω at 5.040 and 6.552 GHz, respectively. Meanwhile, the following two bands have the least point that reached 3.811 and 2.915 Ω at 9.954 and 12.438 GHz, accordingly. In summary, as the real impedance and imaginary refractive index values manifested were greater than or equal to zero, the proposed SQM can be categorised as a passive medium. The electric and magnetic field distributions of three different limits that occurred on the SQM structure for all the resonance frequencies are illustrated in Tables 2 and 3. The basic finding reveals that a strong electric field distribution that occurred at the initial limit of the first four resonance frequencies was present in almost the entire proposed metamaterial structure. The intensity was reduced when the limit increased from 600 V/m to 1200 V/m and 1800 V/m. The first four resonance frequencies had an electric field that was concentrated all over the structure except at the centre (in the vertical direction) of the front metamaterial structure. At 17.838 GHz, the structure had the least distribution for all three limits. Around 95% of the distribution was reduced at the last resonance frequency and just a slight phenomenon occurred at the centre of the design. On the other hand, the SQM had a much lower magnetic field distribution for all three limits, for instance, at 20, 50 and 100 A/m. The magnetic response is created when the wave propagates through the split ring resonator metamaterial, where its magnetic field induces current in the rings. The 20 A/m magnetic field limit had the highest distribution compared to the other limits which concentrated on the outer rings and slowly shifted to the centre when the resonance frequencies moved to higher bands. At each resonance frequency, the magnetic field distribution moderately reduced when the limit increased. Overall, the highest limit possesses less exposure of both electric and magnetic field distribution for all resonance frequencies. Nevertheless, we found a popular question associated with the exposure of these distributions on the surface of Rogers RT6002 material that arose from these findings. Metal-composed materials predominantly have a free electron within their physical structure. Although the free electron is absent in any dielectric substrate, the distributions still take place on its surface. We speculate that this might be due to the delocalisation of the electron oscillation present in metal and substrate materials. This enables both phenomena to occur on the substrate material.

**Table 2:**
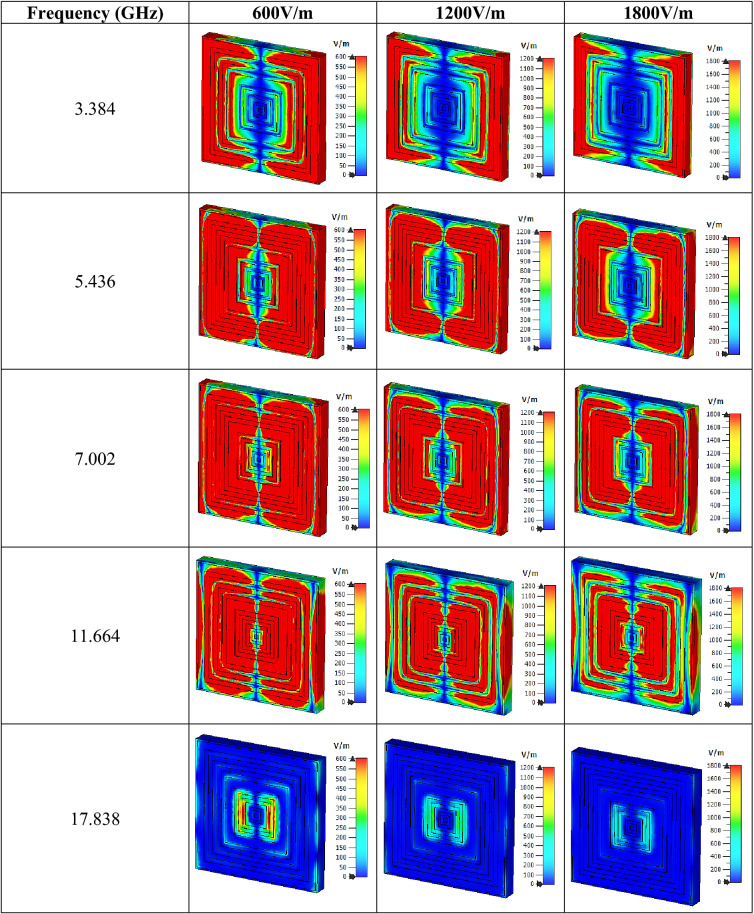
Electric field distribution of the proposed metamaterial design for three field strengths.

**Table 3:**
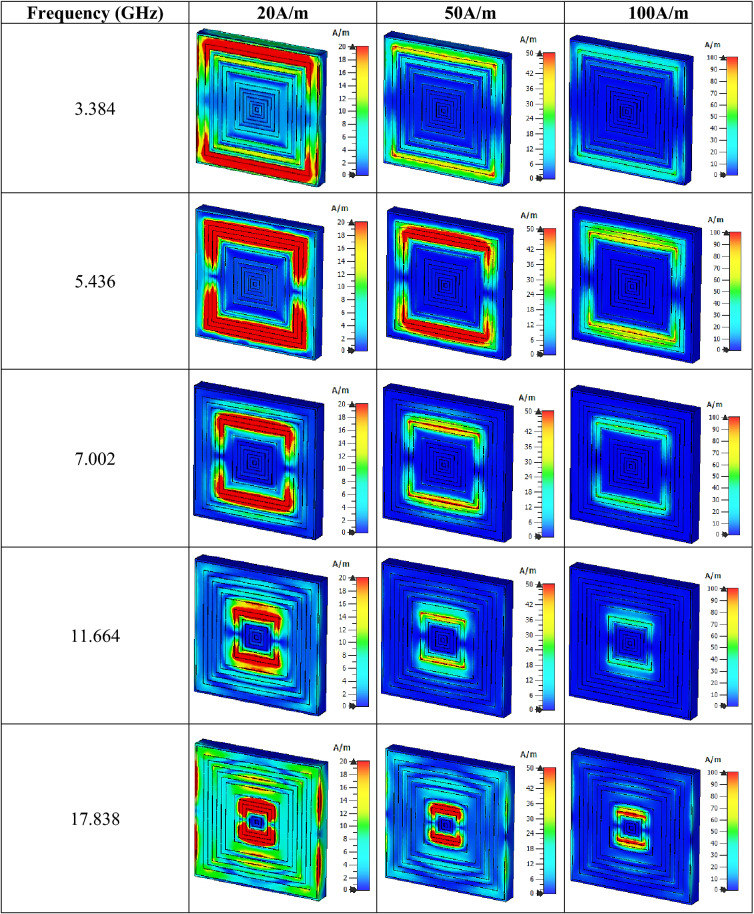
Magnetic field distribution of the proposed metamaterial design for three field strengths.

Finally, the compactness and effectiveness of the SQM can be calculated using the effective medium ratio (EMR) as shown in Eq. (5). The formula is expressed as wavelength per dimension of the metamaterial design, from which the outcome must be more than 4 as an ideal value. If this complies with any metamaterial design, then the results obtained for permittivity and/or permeability will be below zero. The proposed SQM unit cell has a maximum EMR value of 6.33 at the first resonance frequency, which was reduced at the subsequent frequencies.

5 $$ EMR = Wavelength \left( \lambda \right)/Unit\, cell\, length \left( L \right). $$

## SQM design parametric study

The performance comparison between diverse design parameters and constraints are necessary to clearly see the relationship between them. This parametric study allows us to nominate parameters for evaluation, specify design constraint, define the parameter range, and analyse the results of each parameter variation. Several constraints were analysed in this work such as different square-shaped metamaterial design structures, various types of substrate material, waveguide port analysis, and selection of array metamaterial cells. In the end, these simulations help to refine the parameters or design constraints until an optimum metamaterial design was achieved for the stated application.

### Metamaterial design analysis

**Figure 4:**
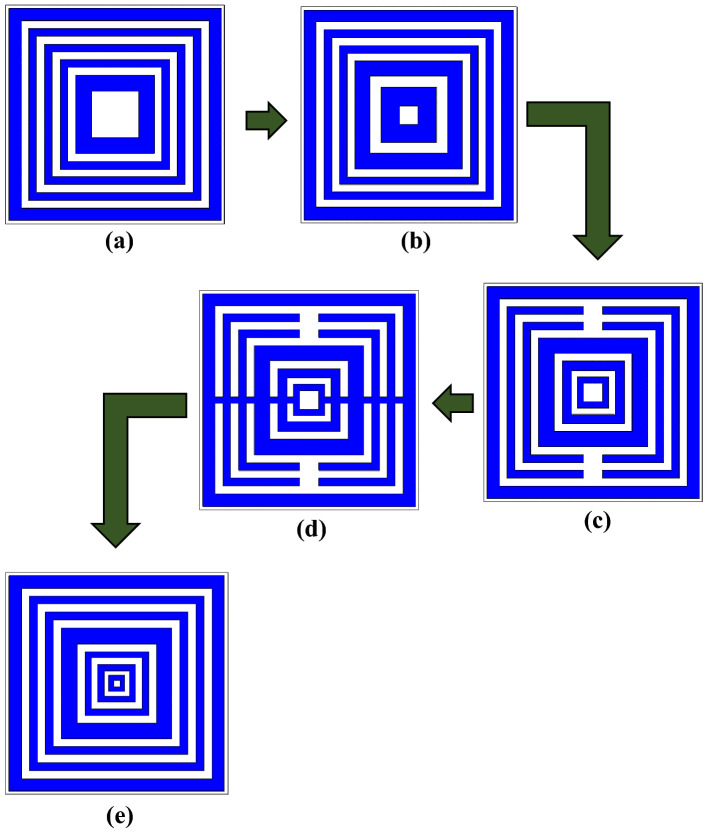
Several square-shaped metamaterial structures: (**a**) DS 1, (**b**) DS 2, (**c**) DS 3, (**d**) DS 4, (**e**) SQM.

In a numerical simulation, the metamaterial construction as illustrated in Fig. 4a–e plays a vital role in obtaining the desired electromagnetic properties. In the preliminary simulation process, we estimated that the resonance frequencies shifted to lower bands when using square-shaped metamaterial design. Besides that, it also performs well and gives good outcomes for the selected frequency range. It is worth discussing the fact that the square-shaped split-ring resonators (SRR), generally induce an electromagnetic force around the structure. Hence, this allows the current to flow under the influence of the external magnetic field, from one ring to another between the inter-ring spacing. Together, the present findings confirm that the square-shaped SRR structure exhibits a lower resonance frequency and has a higher current distribution. Moreover, the composite structure of the square SRR metamaterial possesses a supplementary capacitive coupling and helps manifest a strong resonance behaviour. Hence, several square-shaped metamaterial design (DS) structures were selected for this parametric study as shown in Fig. 4a–e. All the designs in this study have the same substrate material and dimension. There were few apparent differences visible in each design structure, for example, gaps between the rings, subtraction and addition of copper bars, and the number of square SRRs. For the current work, it is sufficient to point out that the number of square rings selection was performed by utilising the trial and error method. In addition, this basic method could simply refer to various simulation analyses with different designs being carried out repeatedly until the optimised design is obtained. Hence, this simple method manifests superior outcomes.

**Figure 5:**
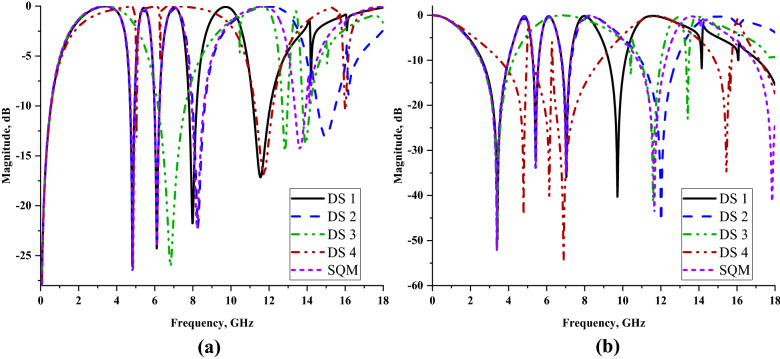
Reflection and transmission coefficients of several square metamaterial structures: (**a**) S11, (**b**) S21.

Figure 5a,b illustrate the reflection and transmission coefficient results of the selected square-shaped metamaterial designs. The planned comparison reveals that nearly all the metamaterial designs gained completely similar transmission coefficient results except DS 3 and SQM designs. The comparison of S21 results indicates that the designs produce one triple, three quadruple, and one quintuple resonance frequencies. The first three resonance frequencies of DS 1, DS 2, and SQM designs manifested nearly similar values at the S- and C-bands with magnitude values of more than − 15 dB. Meanwhile, DS 2 and SQM had the fourth resonance frequency at X-band with slight differences between them but had acceptable magnitude values. The DS 1 and DS 2 designs showed slight differences where the fourth resonance frequency shifted to a higher band when the number of square SRRs was increased. Meanwhile, if the subtracted bar applied in the design as illustrated in Fig. 4c, then the number of resonance frequencies was reduced to two. However, when the additional rectangular bar is added in the design, then the resonance frequencies slightly returned to the normal track. We have verified that using square SRR metamaterial designed in a particular way can obtain the desired resonance frequencies. One of the major concerns in finding the acceptable design was the space limitation in any application fields. Since the demand for miniaturized devices has increased tremendously in the past few years, we face difficulties in designing a compact metamaterial design and at the same time maintain the resonance frequencies of more than four. Furthermore, an important question associated with this problem is how small an author can design a metamaterial structure for the stated application field. Besides that, at this stage of understanding, we believe that smaller sized metamaterial designs have restriction in manifesting lower and more than two resonance frequencies. The initial study of the SQM construction supports the theory above that the smaller sized metamaterial manifests less than two resonance frequencies. Hence, the smallest metamaterial design size that could manifest the desired resonance frequencies, which is 14 × 14 mm^2^, was adapted in this research investigation.

### Substrate material analysis

**Figure 6:**
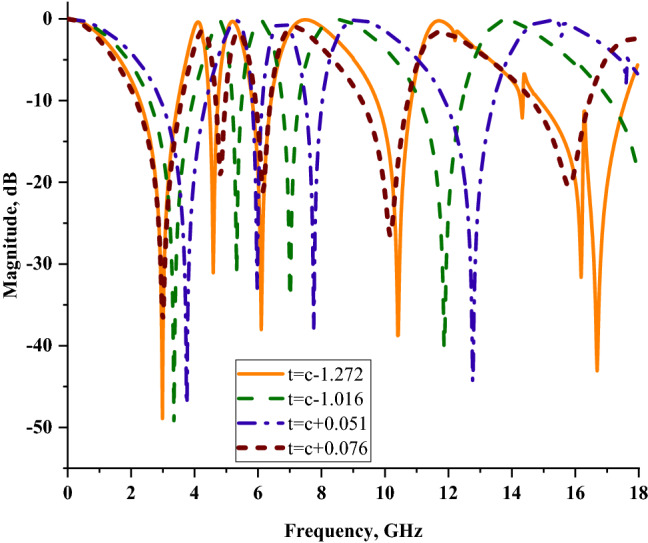
Transmission coefficients (S21) of several substrate materials utilising similar SQM design.

Several substrate materials were selected for this case study such as Rogers RO3006, Rogers RO4003, Rogers RT5880, and FR-4 with the thickness (t) of 0.252, 0.508, 1.575, and 1.6 mm, respectively. Besides that, the substrate materials have dielectric constants of 6.5, 3.55, 2.2, and 4.3, respectively. Figure 6 illustrates the transmission coefficient results of these substrate materials that were manifested by using similar SQM design structure. From the results, it is clearly visible that few substrate materials manifested superior performance than RT6002 material. During the selection process, the stated substrate materials in this analysis were eliminated at the early stage due to several factors. The first reason is the price constraint which led to the elimination of Rogers RO3006. Although this substrate material manifested six resonance frequencies with the smallest substrate thickness, it is more expensive than other substrate materials. Meanwhile, the FR-4 substrate material exhibited the second best outcomes in this analysis. The comparison of this material with RT6002 revealed that the proposed substrate material had slightly higher magnitude values and possessed smaller substrate thickness (c). The Rogers RO4002 and Rogers RT5880 substrate materials exhibited four resonance frequencies at similar frequency bands. Therefore, the Rogers RT6002 substrate material was considered the best among the rest of the stated materials. From the discussion above, a key finding emerges that the dielectric constant values of the substrate materials give a significant contribution to obtain distinct results. Each resonance frequency that the metamaterial structure gained was dominated by these dielectric constant values. In cases where this value was higher, the manifested resonance frequencies shifted to lower bands. Besides that, when the dielectric constant escalated, it influenced the capacitance value at the ground and radiating element at the interval.

### Waveguide port axis analysis

**Figure 7:**
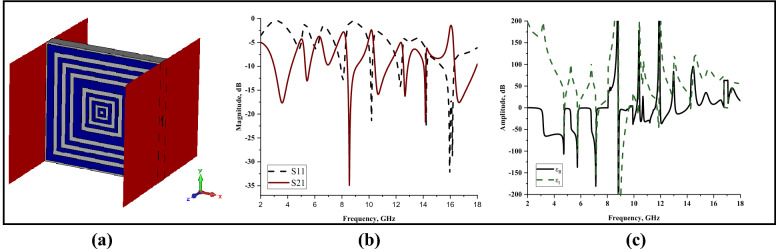
x-axis wave propagation: (**a**) Simulation setup, (**b**) S11 and S21, (**c**) Permittivity.

**Figure 8:**
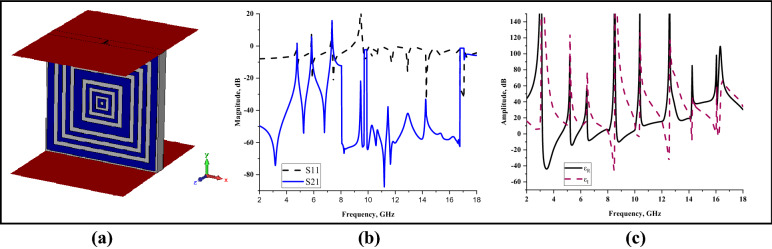
y-axis wave propagation: (**a**) Simulation setup, (**b**) S11 and S21, (**c**) Permittivity.

Besides z-axis wave propagation, the proposed metamaterial design was simulated in x- and y-axes to analyse the changes in the outcome. Figures 7a–c and 8a–c represent the simulation setup of the two different wave propagations and extracted scattering and effective medium parameters by utilising similar methods and techniques as the proposed SQM design. This analysis led to a clear conclusion that both the wave propagations do not manifest the desired resonance frequencies. Furthermore, the resonance frequencies were either manifested with less than − 15 dB magnitude values or unsuitable resonance curve. However, both methods exhibited acceptable permittivity values for the whole frequency range. Meanwhile, better results were achieved when simulating in z-axis wave propagation, for both scattering or effective medium parameters. Hence, these two wave propagations were discarded due to the deficiency of resonance frequencies and unique characteristics.

### Metamaterial array structure analysis

**Figure 9:**
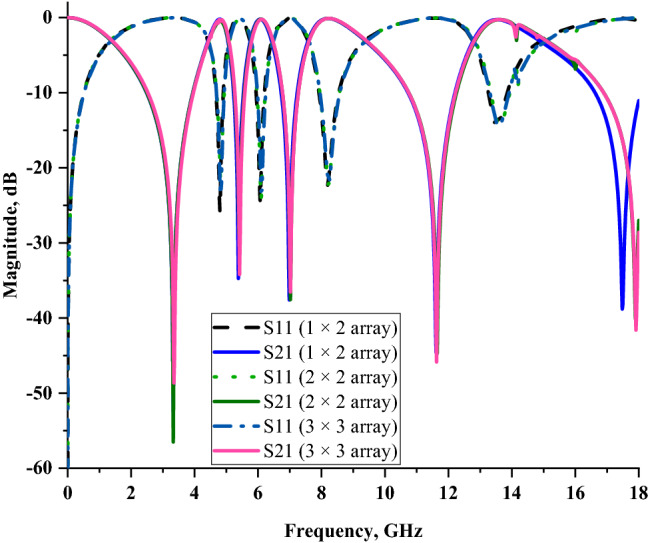
Comparison of reflection and transmission coefficient results of SQM array cells.

We performed a few sets of array cell simulations, such as 1 × 2 (14 × 28 mm^2^), 2 × 2 (28 × 28 mm^2^), and 3 × 3 (42 × 42 mm^2^) for this parametric study. Due to the size limitation, the smaller sized array cells were chosen to perform the comparison. As the unit cell metamaterial is impossible to work independently to exhibit peculiar electromagnetic properties, thus the analysis of array cells was required. Based on the method discussed in the “Materials and methods” section, the numerical simulation for array cells was successfully performed. For this case study, we analysed the data collected from this simulation in one comparison plot as reflected in Fig. 9. All the metamaterial array cells manifested almost similar S21 resonance frequencies except for the fifth resonance for 2 × 2 and 3 × 3 array cells. The outcomes illustrate that the increment of rows and columns in the array structure did not seem to have major impacts on the scattering parameter results. Approximately less than 3% of dissimilarities occurred between each array structure. Regarding the limitation of the metamaterial size, several restrictions need to be adhered to during the selection of array cell design. From this standpoint, with mean dissimilarities of 1.065% when compared to the unit cell, the 1 × 2 array metamaterial design was chosen. Furthermore, with a compact size, the proposed array metamaterial is more convenient and not insignificant for the application field.

## Comparison of previous studies

Table 4 demonstrates the comparison of previous research studies with the proposed SQM design. All the studies claimed that the metamaterial structure was only for single, quadruple, and quintuple resonance frequencies application. Besides that, the previous reference papers analysed manifested negative refractive index, double-negative, and left-handed electromagnetic characteristics. A left-handed characteristic is commonly known as superior electromagnetic property as successfully claimed in Ref.^23^ and SQM design. However, Ref.^23^ has quadruple resonance frequencies at S-, C-, and Ku-bands only. Besides that, the proposed SQM design possesses slightly thinner substrate material and the overall dimension was compact in size. Therefore, the proposed SQM design has leverage over the design in the stated reference. Meanwhile, Refs.^21,22^ exhibited double negative characteristics by utilising distinct substrate materials, namely FR-4 and Rogers RT5880. However, because this research aims to produce a compact metamaterial design with multi-resonance band application, these references show some setbacks. The references either have the biggest array cell design or exhibited single resonance frequency. Besides that, although Ref.^24^ had the smallest array cell structure, it failed to exhibit left-handed characteristics. Most of the previous research investigations selected copper material to construct the metamaterial design on the surface of substrate material. This is because the cost-effective copper material possessing high conductivity besides luxurious materials such as gold and silver. This investigation reveals that the metamaterial with left-handed characteristic is commonly hard to gain for all types of design structures. The gap between rings and compositions of a distinct number of rings have a significant impact on the exhibited result. Generally, the lower resonance frequencies for the square-shaped metamaterial design demonstrates greater current distribution. Furthermore, the effective natural frequency in the metamaterial design can be tuned by altering the composition of the split ring resonators. Consideration regarding the miniaturization of electronic components and devices has emerged into a current trend among the research community. Therefore, a small-sized square-shaped metamaterial structure was proposed in this investigation. Besides that, this type of metamaterial structure is not proposed in any existing research works. The unique arrangement of the metamaterial structure with distinct ring thickness and gap between the rings are the main reason to obtain the desired resonance frequencies. Furthermore, the proposed SQM design manifested left-handed characteristics at all resonance frequencies except S-band. The SQM design also exhibited an effective medium ratio of more than 4 and can be classified as a passive medium as it has the real impedance and imaginary refractive index values greater than or equal to zero. In a nutshell, the study of multi-resonance frequencies for microwave application with a defined compact and simple metamaterial structure became the aim of this research study.

**Table 4:** Comparison between the proposed metamaterial design and previous studies.

References	Operating frequency (GHz)	Dimension (mm^2^)	Resonance frequencies and band	Characteristic	Substrate
^21^	8–14	UC: 10 × 10	Single (X-band)	Double negative	FR-4
^22^	6–18	AC: 180 × 200	Quadruple (C-, X- and Ku-band)	Double negative	Rogers RT5880
^23^	2–14	AC: 10 × 20	Quadruple (S-, C-, and Ku-band)	Left-handed	FR-4
^24^	1–15	AC: 9.6 × 19.2	Quadruple (L-, X-, and Ku-band)	Negative refractive index	FR-4
SQM design	0–18	AC: 14 × 28	Quintuple (S-, C-, X- and Ku-band)	Left-handed	Rogers RT6002

*UC *Unit cell, *AC *array cell*.*

## Conclusion

This paper presented a compact symmetric square-shaped metamaterial (SQM) structure with quintuple resonance frequencies for microwave applications. Five resonance frequencies with acceptable magnitude values were manifested precisely at 3.384 (S-band), 5.436, 7.002 (C-band), 11.664 (X-band), and 17.838 GHz (Ku-band). Furthermore, the results indicate the significant metamaterial design structure that possesses extraordinary properties was obtained from this study. The proposed SQM design manifests left-handed characteristics for three resonance frequency bands, namely at C-, X- and Ku-bands. These findings provide a potential mechanism for increasing the performance in the proposed application field. Future investigation is necessary to validate the kinds of conclusions by setting more constraint variables during the constructions of the metamaterial design. Generally, manifested resonance frequencies can be used in a wide range of application fields such as communications satellites, weather monitoring, direct broadcast satellite services, radar applications, air traffic control, etc. Meanwhile, the SQM design can be applied in any stated applications due to the metamaterial possessing unique electromagnetic properties. In a nutshell, the proposed metamaterial design produces better performances by utilising unique substrate material as it possesses left-handed characteristic for almost all the resonance frequencies and meets the size constraint limitation compared to the existing research works.
